# Intestinal obstruction, obturator hernia, and/or colonic neoplasms: a case study

**DOI:** 10.1093/jscr/rjad583

**Published:** 2023-10-21

**Authors:** Zhaofang Jin, Jianjun Lai

**Affiliations:** Outpatient Department of Surgery, Provincial Hospital Affiliated to Shandong First Medical University, No. 324, Jingwu Road, Huaiyin District, Jinan City, Shandong Province 250021, China; Outpatient Department of Surgery, Provincial Hospital Affiliated to Shandong First Medical University, No. 324, Jingwu Road, Huaiyin District, Jinan City, Shandong Province 250021, China

**Keywords:** intestinal obstruction, obturator hernia, colonic neoplasms, mesh patches

## Abstract

Occlusive hernias are rare and difficult to diagnose. We present an extraordinary case of simultaneous occurrence of an obturator hernia with colon cancer. An 86-year-old woman arrived at the hospital after ˃2 weeks of abdominal pain, nausea, vomiting, and constipation. The computed tomography axis map showed that part of the right lower abdominal small intestine had intruded into the femoral triangle through the obturator, which was diagnosed as an obturator hernia. When the abdominal cavity was opened for herniorrhaphy, a 4 × 4 cm colon mass was observed. Only herniorrhaphy was performed, without any complications. At present, there has been no report of the coexistence of occlusive hernia and colon cancer; the main symptoms are intestinal obstruction, nausea, vomiting, and constipation. The decision whether the tumor should be removed simultaneously with herniorrhaphy and/or a mesh patch.

## Introduction

An obturator hernia is a protrusion of an organ/tissue through the obturator canal. It is commonly known as “little old lady’s hernia,” as they typically affect older, thin female patients [1]. They are rare and difficult to diagnose clinically [2], accounting for 0.05–1.4% of all abdominal wall hernias [3]. Diagnosis is often delayed, and presentation varies with symptoms such as bowel obstruction and pain in the groin or medial thigh [4].

One of the most aggressive types of cancer is colorectal or colon cancer. It is the third most common cancer and a significant cause of morbidity and mortality worldwide [5]. We report a case of an obturator hernia with colon cancer, which has not been reported previously.

## Case report

An 86-year-old female complained of constipation with abdominal pain, nausea, and vomiting for ˃2 weeks. The patient had defecated once, after conservative treatment with an enema in a local hospital, with less abdominal pain and no nausea or vomiting; hence, she was admitted to our hospital for further diagnosis and treatment. The patient’s body temperature was 36.8°C, pulse rate was 87 beats/min, respiration rate was 18 breaths/min, and blood pressure was 125/76 mmHg. The patient was conscious and had a thin appearance, and physical examination indicated that the abdomen was soft, and without tenderness and rebound pain. Routine blood examination showed that hemoglobin was 87 g/L and hematocrit was 28.3%. Abdominal computed tomography revealed the right lower abdominal small intestine protruding into the femoral triangle through the obturator; the upstream intestine was distended and expanded, and multiple fluid levels were observed in the intestinal lumen. Multiple lymph nodes were noticed in the abdomen and retroperitoneum; the large ones had a diameter of ~1 cm. Uneven thickening and enhancement of the local ascending colon were noted.

**Figure 1:**
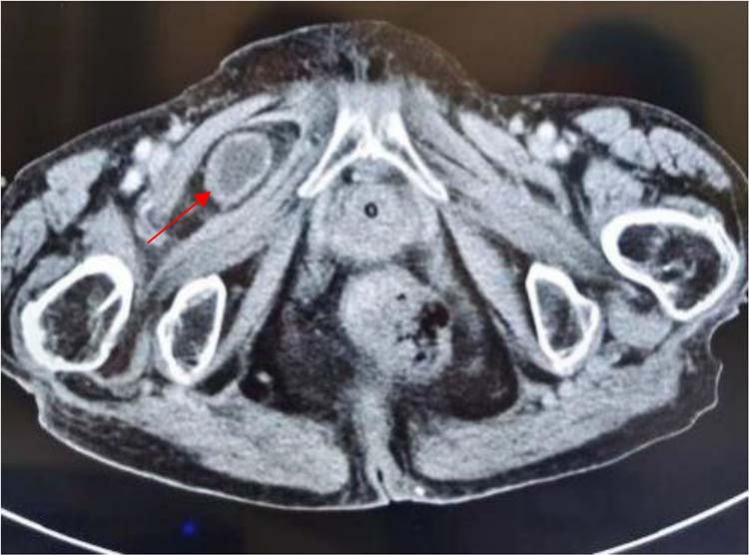
Computed tomography axis map showing the small intestine entering the femoral triangle (red arrow).

**Figure 2:**
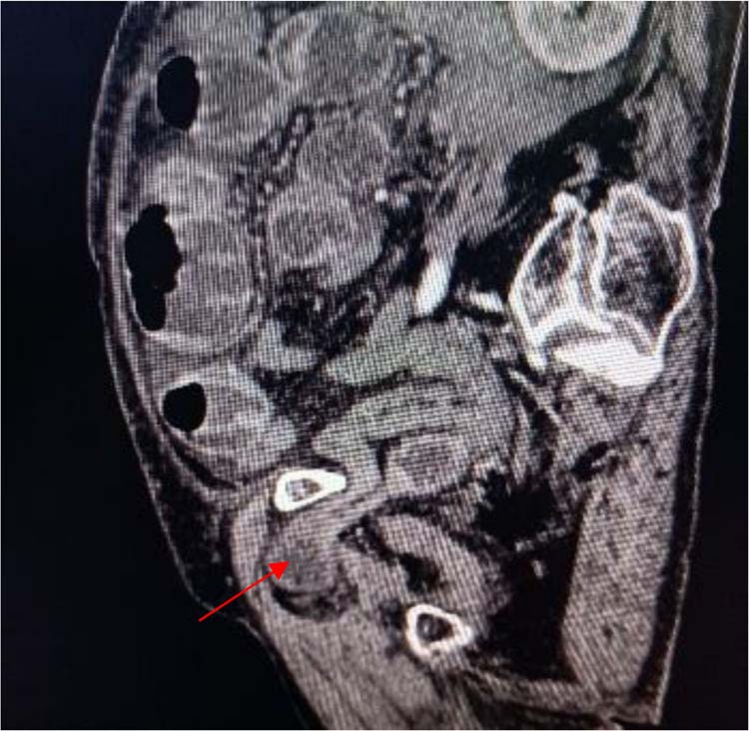
Sagittal view shows a right obturator hernia (indicated by red arrow).

**Figure 3:**
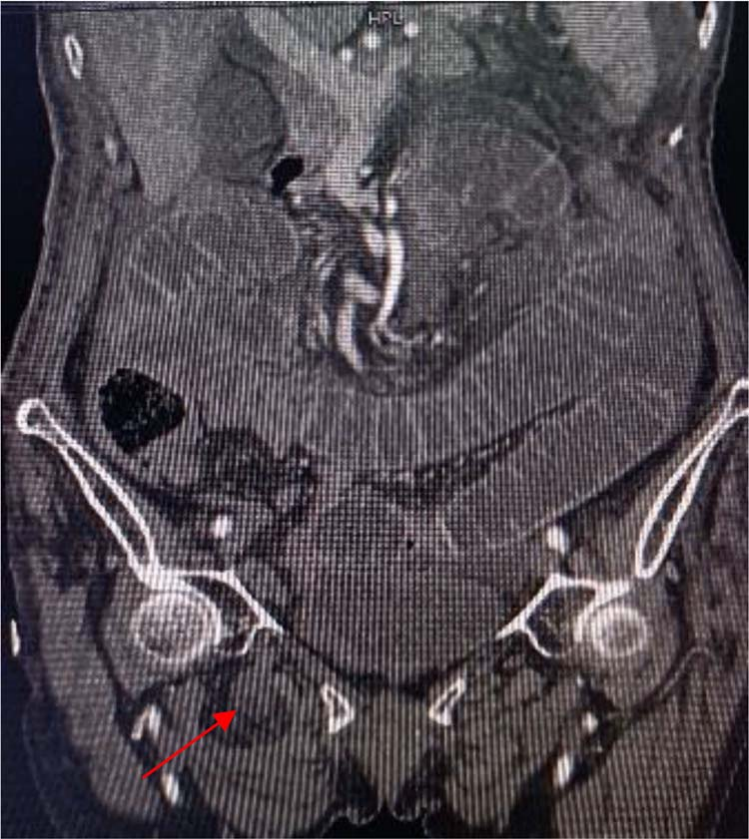
Coronal view shows right obturator hernia entering the intestinal canal (indicated by red arrow).

**Figure 4:**
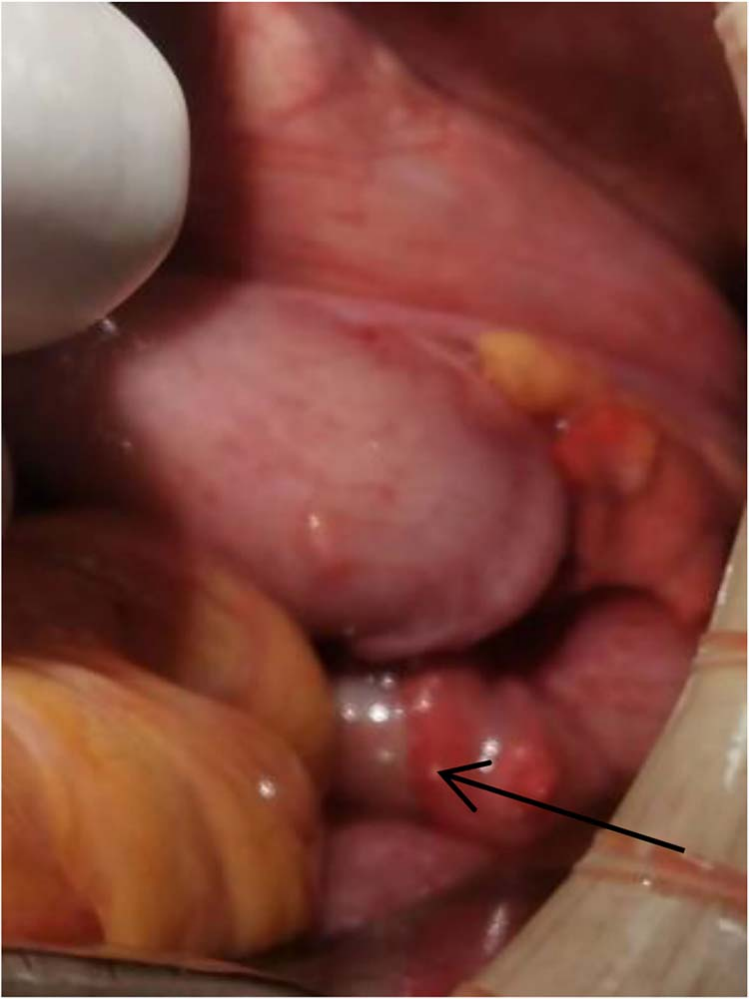
Tumor (indicated by black arrow) on the ascending colon observed during operation.

### Surgical procedures

Laparoscopic exploratory laparotomy and obturator hernia repair were performed. A 4 × 4 cm thick hard tumor was observed in the ascending colon near the liver curve, involving serosa, and the ascending colon was partially blocked. A total of 80 cm away from ileocecum, the intestine entered from the right obturator hernia, causing obstruction and expansion of the proximal intestinal canal, congestion, and edema of the intestinal wall. We aspirated the ascites, loosened the adhesion around the obturator hernia ring, and returned the small intestine back into the abdominal cavity. It was observed that the intestinal wall of the hernia section was ~4 cm, necrotic, and broken, and the intestinal contents were flowing out, the intestinal defect was immediately closed, the abdominal cavity was flushed with normal saline, and the peritoneum at the obturator was intermittently sutured and repaired. A median abdominal incision of ~12 cm was used to cut each layer of the abdominal wall, enter the abdomen, protect the incision, lift out the intestine at the breach, place the aspirator in the intestinal lumen, aspirate large amount of intestinal contents, and temporarily close the breach. The abdominal and pelvic cavities were rinsed again with plenty of normal saline. With the consent of the patient’s family, a double-cavity ileostomy was successfully performed.

## Discussion

To date, the concurrent occurrence of closed hernia and colon cancer has not been reported. This case is the only confirmed case of this type, although the tumor was not removed. We discussed the causes of intestinal obstruction, the surgical methods used, and the use of surgical mesh patches.

The patient was admitted due to intestinal obstruction. Some experts believe intestinal obstruction is caused by intestinal tumors and that obturator hernias are related to them, whereas others propose the underlying cause is a combination of an obturator hernia and colon cancer. We believe the latter is correct because first, our patient was an older woman who was prone to having an obturator hernia due to repeated vaginal delivery and low weight; second, she occasionally complained of thigh pain. During the surgery, it was confirmed that the small intestine had entered the femoral triangle, causing thigh pain; and third, a colonic tumor was discovered during the operation, which caused partial blockage of the ascending colon, the small intestine had extended into the femoral triangle, causing partial necrosis of the small intestine. Regarding the surgical method, some surgeons believe that obturator hernia repair should be performed simultaneously with colon tumor resection, whereas others support performing only the obturator hernia repair. During the operation, the family members were informed of this fact; they agreed, and only obturator hernia repair was conducted, which we preferred. The reasons are as follows: the patient was 86 years old, thin, had a hemoglobin of 87 g/L, and could not have tolerated simultaneous resection. In addition, she had a severe chest infection, abdominal infection, intestinal wall edema, and expansion caused by intestinal obstruction, and an ascending colon tumor. If the obturator hernia repair was performed at the same time as the colon tumor resection, there might have been a risk of leakage of the intestinal anastomosis and aggravation of the ascending colon obstruction. Therefore, major surgery for concurrent removal of the ascending colon tumor was not considered.

There are conflicting opinions about the use of mesh patches. We prefer not to use mesh patches. Laparoscopic mesh placement is the most appropriate treatment, although only under the right patient conditions [6]. Hosoi *et al.* [7] believe there is a risk of serious contamination in placing mesh in contaminated cases, therefore, the mesh does not need to be placed, and it is only applicable for clean cases. Compared with clean cases, the risk of postoperative occurrence in contaminated cases after abdominal hernia repair with mesh significantly increases [8]. Our patient had a severe abdominal infection and was not suitable for mesh patch use.

This is the first reported case of concurrent occlusive hernia and colon cancer. The main symptoms were intestinal obstruction, including nausea, vomiting, and constipation. The decision whether the tumor should be removed simultaneously with herniorrhaphy, and/or using the mesh patch during herniorrhaphy depends on the comprehensive situation of the patient.

## Data Availability

Data availability is not applicable to this article as no new data were created or analyzed in this study.
